# Can Acting Out Online Improve Adolescents’ Well-Being During Contact Restrictions? A First Insight Into the Dysfunctional Role of Cyberbullying and the Need to Belong in Well-Being During COVID-19 Pandemic-Related Contact Restrictions

**DOI:** 10.3389/fpsyg.2021.787449

**Published:** 2022-01-10

**Authors:** Jan S. Pfetsch, Anja Schultze-Krumbholz, Katrin Lietz

**Affiliations:** Educational Psychology, Technische Universität Berlin, Berlin, Germany

**Keywords:** cyberbullying, need to belong, emotion regulation, loneliness, well-being, contact restrictions, adolescence

## Abstract

Connecting with peers online to overcome social isolation has become particularly important during the pandemic-related school closures across many countries. In the context of contact restrictions, feelings of isolation and loneliness are more prevalent and the regulation of these negative emotions to maintain a positive well-being challenges adolescents. This is especially the case for those individuals who might have a high need to belong and difficulties in emotional competences. The difficult social situation during contact restrictions, more time for online communication and maladaptive emotion regulation might lead to aggressive communication patterns in the form of cyberbullying perpetration. In an online study with *N* = 205 adolescents aged 14–19 (*M* = 15.83, *SD* = 1.44; 57% girls), we assessed the frequency of online and offline contacts, need to belong, emotion regulation problems, feelings of loneliness, and cyberbullying perpetration as predictors of adolescents’ well-being. In particular, we explored whether cyberbullying perpetration might function as a maladaptive strategy to deal with feelings of loneliness and therefore predicts well-being. This effect was expected to be stronger for those with a higher need to belong and with higher emotion regulation problems. Results of a hierarchical regression analysis revealed that well-being was significantly predicted by less emotion regulation difficulties, less feeling isolated and more cyberbullying perpetration. We also tested whether the need to belong or emotion regulation problems moderated the association between cyberbullying and well-being. While the results for emotion regulation problems were not significant, the moderation effect for the need to belong was significant: For students with a high need to belong, well-being was more strongly related to cyberbullying perpetration than for students with a medium need to belong. For students with a low need to belong, cyberbullying was not significantly associated with well-being. That cyberbullying perpetration predicted well-being positively is rather surprising in the light of previous research showing negative psychosocial outcomes also for cyberbullying perpetrators. The moderation analysis provides a hint at underlying processes: In times of distance learning and contact restrictions, cyberbullying may be a way of coming into contact with others and to regulate loneliness maladaptively.

## Theory

Connecting with peers online to overcome social isolation has become particularly important during pandemic-related school closures and restrictions of social contacts across many countries worldwide. In Germany, schools were closed in spring 2020, partly closed again from mid-December 2020 to June 2021, and additional strict contact restrictions were in place for everyday life. Therefore, digital communication tools gained importance to reach out to and to interact with peers, especially to cope with the fast-changing challenges in the context of the COVID-19 pandemic. However, the use of electronic means of contact also bears the risk of negative behaviors and experiences that benefit from the anonymous and unregulated nature of social media and social online networks. One of these potentially negative behaviors is cyberbullying. In the current study we focus on feelings of loneliness in times of school closures and contact restrictions, the regulation of these negative emotions, and cyberbullying as a strategy for emotion regulation in connection with the well-being of adolescents.

Adolescents normally spend a huge part of their days in school and besides teaching content knowledge, schools also facilitate students with the possibility of intensive social contact to peers. Being in school a considerable time of the day together with other students in classes and recess, schools represent an important socializing context for social-emotional development and contributes to the fulfillment of the need to belong of adolescents (Raufelder et al., 2021). In Germany, for example, students in lower secondary schools would regularly receive 4,502 h of compulsory instruction time in general education (OECD, 2021a, Table D1.1). However, in the school years 2019/2020 and 2020/2021 German schools were completely closed for 64 days in primary, 85 days in lower secondary and 83 days in higher secondary education (OECD, 2021b). This means, that children and adolescents were largely cut off from meeting their peers in person, which may have impacted their well-being, loneliness and social behavior. As the pandemic and related contact restrictions can constitute a fundamental strain, and social support usually provides an important resource for coping with strain, conflicts, and crises (Hurrelmann and Quenzel, 2018), negative emotions, loneliness and emotion regulation problems may occur under these specific circumstances. Externalizing, aggressive behavior may result as a reaction to an imbalance between developmental requirements on the one hand (like pandemic related rules of physical distancing) and individual and social resources on the other hand (like emotion regulation skills and social support), which can be related to negative outcomes for personal individuation and social integration (Hurrelmann and Quenzel, 2018). The present study therefore analyzes the relation of well-being, dysfunctional aggressive behavior, and the need to belong among adolescents under pandemic-related contact restrictions.

### Cyberbullying and Well-Being

Cyberbullying, understood as the repeated, intentional harm of others via digital communication media, has been shown to have increased during the COVID-19 pandemic. In a recent study, Beitzinger et al. (2020) found that in 2017, 12.7% of German students aged 8–21 had been cyberbullied. In December 2020, the number increased to 17.3%. Furthermore, it showed that every 4th student among 13–17-year olds is affected by cyberbullying. Compared to previous years, this represents an increase of 20% during school closures. However, this study is disputed due to methodological weaknesses.

A wide array of potential outcomes of cyberbullying regarding mental and physical health have been investigated in previous years. Cyberbullying is generally negatively related to well-being and psycho-social adjustment (Kowalski et al., 2014; Guo, 2016; Ansary, 2020). Psychological well-being encompasses subjective experience of happiness and life satisfaction (hedonic perspective), and psychological functioning and self-realization (eudaimonic perspective; Ryan and Deci, 2001), thus has affective, cognitive-evaluative, and psychological functioning dimensions (Tennant et al., 2007). In a meta-analysis of 131 studies from around the world that had been published or in press by Fall in 2012, Kowalski et al. (2014) found that cyberbullying perpetration was significantly linked to depression, low self-esteem, anxiety, loneliness, drug and alcohol use, academic achievement and life satisfaction.

Regarding well-being in the context of school closures and distance learning, 25% of German children reported frequent conflicts within families and with friends and more school stress; 65% of children perceive learning during the second lockdown as more stressful than before the pandemic (Ravens-Sieberer et al., 2021). However, studies from other countries such as Xie et al. (2020) and Demkowicz et al. (2021) also indicate that the circumstances of the pandemic do not necessarily cause entirely negative feelings in students if they are combined with behavioral strategies that increase well-being, for example, by maintaining contact with peers and if the situation is perceived as changeable and temporary rather than hopeless. In times of contact restrictions, cyberbullying may be a way of coming into contact with or eliciting reactions from others. Also, resorting to cyberbullying may be a way to solve or carry out conflicts that may otherwise have been carried out in school.

### Emotion Regulation in Adolescents and Cyberbullying

While in early adolescence mood swings and increasing negative emotions are quite prevalent, it is only in late adolescence that affective experiences become more stable (Larson et al., 2002). This shows that adolescents learn stepwise to regulate their emotions and the emergence of emotion regulation—understood as monitoring, evaluating, and modifying the quality, intensity, dynamic and expression of emotions to accomplish one’s goals (Thompson, 1994)—is an important developmental step in adolescence (Philips and Power, 2011). Adolescents become more and more effective and adaptive in the way they modulate positive and negative emotions (Stifter and Augustine, 2019), however, there are considerable differences in emotion regulation competences between persons. Poor psychosocial functioning and impaired social relationships or even externalizing problems like aggressive behavior can be associated with emotion regulation difficulties (Philips and Power, 2011; Bjureberg et al., 2016). Cyberbullying can be seen as one of these externalizing problem behaviors, and it seems to be related to problems in emotion regulation. Emotional management has been shown to be negatively related to cyberbullying perpetration in a meta-analysis including four effect sizes (Chen et al., 2017). For example, in a study about resilience and emotion regulation in adolescents, a subgroup of cyberbully victims reported high negative emotions, emotional problems, and they showed low levels of resilience and positivity (Gianesini and Brighi, 2015). Further, in a study about emotional intelligence, the regulation and use of emotions emerged as a significant negative predictor for being a cyberbully (Baroncelli and Ciucci, 2014). This effect was stable even after controlling for offline bullying and indicates that cyberbullies seem to have emotion regulation problems. Over and above low friend support and family cohesion, impulse control difficulties and lack of emotional clarity positively predicted cyberbullying perpetration (Arató et al., 2021). Additionally, in a structural equation model, the interaction of anger and negative emotion regulation (like other-blame, or rumination) predicted cyberbullying perpetration positively. The effect of anger on cyberbullying was higher for those participants with high negative emotion regulation compared to participants low in negative emotion regulation (Den Hamer and Konijn, 2016). Taken together, previous research shows that experiencing negative emotions, using negative emotion regulation strategies (especially blaming others), and showing emotion regulation problems (especially impulse control difficulties and lack of emotional clarity) are positively related to cyberbullying perpetration.

### Loneliness

Generally, positive emotions decline during adolescence, negative emotions and emotion instability increase (Larson et al., 2002; Rosenblum and Lewis, 2003), and sensitivity to social threats and rejection increases (Goossens, 2018). It is therefore not surprising that loneliness is particularly important in this phase of life.

Loneliness is defined as a subjective state of distress caused by the discrepancy between the desire for and the perception of social relationships (Peplau and Perlman, 1982; Jeste et al., 2020). It is closely related to peer relationships, family cohesion, and self-esteem (Jeste et al., 2020). Loneliness can be linked to a variety of physical and mental disorders, including heart disease, depression, and cognitive impairment (Cacioppo et al., 2010; Lim et al., 2016; Valtorta et al., 2016; Donovan et al., 2017). It is often perceived as a problem of elderly people. However, Victor and Yang (2012) were able to show that loneliness is U-shaped across the lifespan, meaning that loneliness is most common among those under 25 and over 65. While for older people the quality of social relationships offers a protective factor against loneliness, younger generations place more value on their quantity (Victor and Yang, 2012; Nicolaisen and Thorsen, 2017). Interestingly, in both groups the desire for contact with friends has a greater influence on loneliness than actual contact (Nicolaisen and Thorsen, 2017). Further, Child et al. (2017) show that young adults are twice as likely to report lonely and isolated days as late middle-aged adults, despite having larger networks. Possibly the superficial satisfaction of a variety of social contacts is not enough to compensate for feelings of loneliness because the sense of positive support from meaningful peer relationships is lacking to establish well-being in adolescents (Harter, 1985).

While media use was already an important part of everyday life for young people in Germany before 2020, a growing relevance of young people’s media behavior could be observed compared to previous years (Medienpädagogischer Forschungsverbund Südwest (MPSF), 2020), due to closed leisure time activities and distance learning. For communication with friends, messengers such as WhatsApp remain the most popular providers, with 94% of young people reporting that they use them to communicate with others several times a week. 87% of respondents also said they had a WhatsApp group with their class. The platform TikTok showed an increase of 19% in regular use (Medienpädagogischer Forschungsverbund Südwest (MPSF), 2020). Especially considering that the frequency of face-to-face meetings with peers leads to fewer feelings of loneliness (Nicolaisen and Thorsen, 2017), the question remains whether contact via digital media, in the context of part time school closures and contact restrictions, could compensate for feelings of loneliness and how adolescents were able to still feel comfortable in the unfamiliar situation. In the context of contact restrictions, feelings of isolation and loneliness are likely to be more prevalent than usual. In addition to worries and fears, loneliness among youth played a major role during the Corona pandemic: More than one third of the respondents reported that they felt lonely in the current situation (Walper et al., 2021). In addition, 30% of the adolescents still felt socially isolated even after relaxation of the measures (Rauschenberg et al., 2021). To escape these feelings of loneliness and isolation, contact with peers online became especially important for adolescents.

### Need to Belong

Affiliation with others is one of the basic human needs and developing positive social bonds is one of the core developmental tasks in adolescence (Hurrelmann and Quenzel, 2018). The need to belong refers to the need of humans to form meaningful, positive, caring and (temporarily) stable interpersonal relationships. Many theories dedicated to explaining human behavior include the dimension of belonging or relatedness to others (i.e., choice theory, Glasser, 1985; self-determination theory, Ryan and Deci, 2000; also see Baumeister and Leary, 1995, p. 497: psychoanalysis, Freud, 1930; motivation theory, Maslow, 1968; attachment theory, Bowlby, 1969, 1973). According to Baumeister and Leary (1995), the need to belong is innate and of evolutionary relevance, and should be found in every human being, although to varying degrees. The loss of specific relationships may be compensated by other or new relationships. Since the need to belong differs from a mere need for social contacts, a certain level of intimacy needs to be established first, though. Contact restrictions could result in a loss of such intimate relationships, especially outside of family bonds, if they are dependent on contact in person.

Belonging encompasses a number of different domains and is not limited to one context. Family, friends or peers, school, and neighborhood and community have been identified as relevant domains (cf. Jose et al., 2012). Studies examining different domains simultaneously found that the number of domains in which connectedness was perceived was negatively related to emotional stress in adolescents (Libbey et al., 2002). Generally, connectedness was associated with lower levels of stress, depression and anxiety, with connectedness with peers showing especially strong effects in one study among final-year secondary school students (McGraw et al., 2008). Another study with adolescents showed that family and school connectedness were the most strongly related to well-being over the course of 3 years. Also, all domains were related in a reciprocal way, that is, connectedness fostered well-being which in turn was related to increases in connectedness (Jose et al., 2012). Recent studies on distance learning under COVID-19 restrictions also highlighted the importance of perceived relatedness for well-being, such as the study by Holzer et al. (2021) which found a significant positive association of perceived relatedness with positive emotions (and learning-related outcomes) among more than 25,000 adolescents from eight different countries.

The need to belong may not only be satisfied by positive relationships, but could also be met through aggressive means like bullying others (Underwood and Ehrenreich, 2014). Previous studies have shown that the need to belong may be a mechanism behind aggression among children and adolescents. For example, when adolescents feel a strong need to belong, but lack skills in regulating negative emotions, they might be at risk for perpetrating offline bullying and cyberbullying. A limited number of studies have examined this connection between belonging (as an element of basic needs) and cyberbullying perpetration. Mainly, studies have focused on the need to belong in the context of offline bullying and victimization (e.g., Hein et al., 2015; Lam et al., 2015; Menéndez Santurio et al., 2020), satisfaction of basic needs in general and not testing belonging specifically (e.g., Varsamis et al., 2021), or—more extensively—in the workplace (e.g., Trépanier et al., 2013). Hein et al. (2015) examined in their study in Estonia how controlling behavior by (physical education) teachers prevents the needs satisfaction of adolescent students’ basic needs (according to the self-determination theory [SDT] by Ryan and Deci, 2000: autonomy, competence and relatedness) and found that the influence of selected dimensions of controlling behavior on offline bullying was mediated by need thwarting, i.e., more controlling teacher behavior increased need thwarting which in turn was related to increased levels of offline bullying behavior by the respective students. In Hong Kong, Lam et al. (2015) conducted a study, also based on SDT, to identify latent profiles of offline bullying involvement among adolescent students and to determine the role of basic needs as predictors for group membership. They showed that students were less likely to be an offline bully or victim when they reported their need for relatedness to be met by teachers. In their study on the association of basic psychological needs with offline bullying and victimization in the family and at school, Varsamis et al. (2021) only found direct links of school-based fulfillment of basic needs with offline victimization, but not bullying. However, family-based satisfaction of basic needs was associated with lower levels of adolescents’ offline bullying behavior in school.

Regarding cyberbullying, Solomontos-Kountouri and Strohmeier (2021) found that although the need to belong did not generally differ significantly between non-immigrant, first-generation immigrant and second-generation immigrant adolescents in Cyprus, belonging or affiliation was the main and sole motive for first-generation immigrant adolescents to offline bully or cyberbully others. For the other two groups, other motives (i.e., power and anger) were also relevant. In order to be accepted by peers, first-generation immigrant adolescents seem to resort to the use of aggression. This has also been supported by research in the offline context: especially first-generation immigrant adolescents resort to offline bullying behavior in order to achieve acceptance and affiliation among their peers (Fandrem et al., 2009; Strohmeier et al., 2012). Tanrikulu (2015) generally reports an association between an unmet need to belong and cyberbullying perpetration among adolescents in Turkey.

From the reviewed literature it becomes clear that close, caring and mutual connections to others are important for well-being and a successful development, especially in adolescence and that an unfulfilled need to belong could result in aggressive behavior, during times of contact restrictions most likely via electronic means.

### Present Study

The contact restrictions during the COVID-19 pandemic reduced personal contact to mostly online contact. This may have led to an increase in loneliness and need thwarting regarding the need to belong. Since affiliation and connectedness foster positive emotions and reduce emotional stress, the fulfillment of the need to belong might be seen as a protective factor. With the effect of this protective factor being limited now by the forced reduction of social interactions, adolescents (especially those with emotion regulation problems) might now have problems dealing with negative emotions such as loneliness. At the same time, media use has likely increased with it being one of the few means to get in contact with others, but also providing opportunity for negative online behavior. Since an unsatisfied need to belong has been associated with aggressive behavior in previous research, we hypothesized that cyberbullying perpetration might function as a maladaptive strategy to deal with feelings of loneliness and therefore predicts well-being significantly. This effect might be even stronger for those with a higher need to belong and with more emotion regulation problems. We therefore formulated the following hypotheses:

H1: Loneliness and cyberbullying are positively associated with each other.
H2: Cyberbullying significantly predicts well-being.
H3: Emotion regulation problems moderate this relationship of cyberbullying with well-being significantly. The higher the emotion regulation problems, the stronger the relation between cyberbullying and well-being.
H4: Need to belong moderates the relationship of cyberbullying with well-being significantly. The higher the need to belong, the stronger the relation between cyberbullying and well-being.

## Materials and Methods

### Procedure

We conducted an online survey and distributed the link to schools to pass it on to their students. Because of the sensitivity of the covered topic, we restricted the participation to students aged 14 years and older. According to the ethical guidelines and federal legislation adolescents from an age of 14 and older are considered to have the authority to decide independently about their participation in the current research project.

Participants were informed comprehensively about the anonymous, free participation, the planned use of data and their right to end the participation without negative consequences. They explicitly agreed to these study conditions by ticking their approval at the beginning of the online survey. In this way, informed consent was assured according to the ethical guidelines and federal legislation. The study took place outside of online classes and during students’ leisure time.

### Sample

The university’s school office was consulted for recruiting the sample. As part of the General Student Advisory Service, this office works at the interface between schools and the university and coordinates the cooperation with schools. Since it is in contact with numerous partner schools, we were able to ensure the broadest possible reach by having the head of the school office send the link for this study via email to all partner schools.

Two-hundred-and-five adolescents aged 14–19 years (*M* = 15.83, *SD* = 1.44) participated in our online study with the majority of participants being 14, 15, or 16 years old (> 20% each). Of the participants, 57% were girls and 31% were boys, 12% preferred not to disclose their gender. Participants mostly attended high academic track schools (75% Gymnasium), about 20% visited polyvalent schools (Gemeinschaftsschule), the rest visited low academic track schools (3% Integrierte Sekundarschule), vocational schools (2% Berufsschule/Oberstufenzentrum) or another school type (1%). Participants were 7th–13th graders, with the majority being in 9th grade, 10th grade or 12th grade (> 25% each). As the study was conducted between mid-March and the beginning of April 2021 while most schools were closed during the second lockdown in Germany, only 29 of the students (14%) had visited any sort of classroom teaching in the 4 weeks prior to our study (e.g., to receive special education or technical support for distance learning).

### Measures

The dependent variable *well-being* was assessed with an adapted 7-item short version of the Short Warwick-Edinburgh Mental Well-Being Scale (SWEMWBS; Tennant et al., 2007; German translation by Bachinger and Lang, 2013). We asked participants to indicate how often they had felt the respective ways asked in the specific items (e.g., “I felt relaxed.”) during the previous 4 weeks (1 “never” to 5 “always”), Cronbach’s α = 0.86. We exchanged one item about feeling close to others with feeling well overall, because the overlap with belonging was too big.

The independent variables were measured as follows. For *cyberbullying* we used the perpetration scale of the Direct and Indirect Cyberbullying and Cybervictimization scales (DICC; Pfetsch, 2013). It comprises eight behavior-based items on negative online behaviors, e.g., “I sent mean messages to someone in order to hurt them (e.g., over Instagram, Facebook, WhatsApp).” Participants were asked to indicate how often they had done these things intentionally during the past 4 weeks (1 = *never* to 5 = *almost daily*), Cronbach’s α = 0.92. The *need to belong* was assessed with an adapted version of the Leary Need to Belong Scale (Leary et al., 2006; German translation by Hartung and Renner, 2014) in which we used seven of the originally 10 items, e.g., “I seldom worry about whether other people care about me.” (inversely coded). Participants were asked to indicate in how far the statements apply to them (1 = *not at all* to 6 = *absolutely*), Cronbach’s α = 0.70. *Emotion regulation problems* were measured using the subscales “Impulse Control Difficulties” (*low impulse control*), “Limited Access to Effective Emotion Regulation Strategies” (*limited strategies*), and “Lack of Emotional Clarity” (*lack of clarity*) from the Brief Version of the Difficulties in Emotion Regulation Scale (DERS-16; Bjureberg et al., 2016; German translation by Gutzweiler and In-Albon, 2018). This resulted in nine items (1 = *almost never* to 5 = *almost always*), e.g., “When I am upset, I become out of control,” Cronbach’s α = 0.89. We excluded one item from the strategies subscale because there was too much conceptual overlap with a different subscale. For *loneliness* we adapted the Revised University of California Los Angeles Loneliness Scale (UCLA-R LS; Russell et al., 1980; German translation by Döring and Bortz, 1993). From initially twenty items (10 for each subscale “Subjective Feelings of Loneliness” and “Feelings of Social Isolation”) we selected 10 items based on the psychometric properties presented by Döring and Bortz (1993) to form the two subscales *Feelings of Isolation* (e.g., “I feel isolated from others.”) and *Lack of Proximity* (e.g., “I have got people who are close to me.”—inversely coded), Participants were asked to indicate in how far the statements applied to them during the previous 4 weeks (1 = *not at all* to 5 = *completely*), Cronbach’s α = 0.84 and 0.81. For the assessment of *contact frequency*, we developed new measures, based on comparable items from Guglhör-Rudan et al. (2007). To assess the *frequency of direct contact*, we asked participants to indicate how often they had met close family, relatives, partner and children, roommates, friends, classmates, and others in person during the past 4 weeks (1 = *never* to 6 = *almost daily/daily*). As expected for adolescents, nearly all met close family on an almost daily or daily basis (*M* = 5.79, *SD* = 0.82, *Mdn* = 6), but their friends (*M* = 3.15, *SD* = 1.39, *Mdn* = 3) and classmates (*M* = 2.81, *SD* = 1.49, *Mdn* = 3) far less often in person. To assess the *frequency of digital contact*, we asked participants to indicate how often they had telephoned in audio/video with close family, relatives, partner and children, roommates, friends, classmates, and others during the past 4 weeks (1 = *never* to 6 = *almost daily/daily*). Contrary to direct contacts, participants talked to their close family on telephone only rarely (*M* = 2.82, *SD* = 1.90, *Mdn* = 3), but they spoke more often to their friends (*M* = 4.08, *SD* = 1.72, *Mdn* = 5) and classmates (*M* = 3.88, *SD* = 1.69, *Mdn* = 4). All scales were computed based on mean scores across the respective items.

### Data Analyses

We computed correlations for the description of the bivariate relations and a hierarchical regression analysis using SPSS to predict well-being regressed on need to belong, emotion regulation problems, loneliness, and cyberbullying, with age, gender and contact frequency in digital and direct contexts being controlled for. Subsequently, we tested whether the need to belong or emotion regulation problems moderated the association between cyberbullying and well-being using the PROCESS macro in SPSS. In an exploratory analysis, we analyzed the moderating effect of both loneliness subscales isolation and proximity on the association between cyberbullying and well-being using the PROCESS macro in SPSS.

The assumptions for the statistical analyses were tested (Field, 2013). Because not all variables were normally distributed, we conducted Spearman rho correlations for the bivariate relations. Concerning the regression analyses, we tested the following assumptions: significant bivariate relations with well-being were tested, but we also included variables with non-significant relation for content-related reasons, e.g., age or contact frequency. Multicollinearity was not indicated by values of tolerance (all > 0.2, Menard, 1995) and variance inflation factor (all < 0.10, Myers, 1990), but slight multicollinearity was indicated by a condition index of 59. No auto-correlation was indicated by a Durbin Watson statistic of *d* = 2.2 because it was between 1 < *d* < 3. Homoscedasticity was indicated by a constant variance in the scatter plot of standardized predicted values vs. standardized residuals. Normal distribution of the residuals was indicated by only small deviations from the diagonal line of the normal P-P- plots. In sum, assumptions were generally met, and statistical analyses could be performed without constraints.

## Results

Bivariate correlation analyses (see Table 1) show that well-being is significantly negatively related to the need to belong, emotion regulation problems and loneliness. Surprisingly, cyberbullying was positively, but not significantly related to well-being. Concerning hypothesis 1, the loneliness subscale “isolation” was significantly correlated with cyberbullying, but this association was negative and not positive. The loneliness subscale “proximity” was negatively, but not significantly related to cyberbullying and, in sum, hypothesis 1 has to be rejected.

**TABLE 1:** Spearman rho correlations between interval variables.

	Well-being	Direct contact frequency	Digital contact frequency	Need to belong	ER—Lack of Clarity	ER—low impulse control	ER–limited strategies	Loneliness– isolation	Loneliness– proximity	Cyberbullying
Well-being	1	0.001	0.16	−0.37***	−0.56***	−0.46***	−0.69***	−0.67***	−0.52***	0.15
Direct contact frequency	0.001	1	0.59***	0.09	–0.07	–0.01	–0.01	0.01	–0.10	0.11
Digital contact frequency	0.16	0.59***	1	0.04	–0.14	–0.03	–0.08	–0.13	−0.16*	0.13
Need to belong	−0.37***	0.09	0.04	1	0.16	0.16	0.30***	0.40***	0.08	–0.05
ER—lack of clarity	−0.56***	–0.07	–0.14	0.16	1	0.42***	0.62***	0.44***	0.32***	–0.01
ER—low impulse control	−0.46***	–0.01	–0.03	0.16	0.42***	1	0.60***	0.28***	0.22***	0.13
ER—limited strategies	−0.69***	–0.01	–0.08	0.30***	0.62***	0.60***	1	0.51***	0.31***	0.01
Loneliness—isolation	−0.67***	0.01	–0.13	0.40***	0.44***	0.28***	0.51***	1	0.59***	−0.21*
Loneliness—proximity	−0.52***	–0.10	−0.16*	0.08	0.32***	0.22***	0.31***	0.59***	1	–0.14
Cyberbullying	0.15	0.11	0.13	–0.05	–0.01	0.13	0.01	−0.21*	–0.14	1

ER, emotion regulation. *p < 0.05, ***p < 0.001; N = 137—171.

The hierarchical regression analysis for the dependent variable well-being is displayed in Table 2. Predictors were included simultaneously in four steps: First, age, gender, and contact frequency in digital and direct contexts were included to serve as control variables. Second, need to belong and emotion regulation problems were included as indicators of interpersonal difference. Third, loneliness was included to analyze the effect of affective experiences under circumstances of contact restrictions. Forth, cyberbullying was included as predictor to understand its role related to positive well-being.

**TABLE 2:** Hierarchical regression analysis for the prediction of well-being.

	Step1			Step 2			Step 3			Step 4		
	*B*	*SE*(*B*)	β	*B*	*SE*(*B*)	β	*B*	*SE*(*B*)	β	*B*	*SE*(*B*)	β
Constant	3.07	0.77		4.89	0.63		5.43	0.60		4.84	0.64	
Age	–0.05	0.04	0.10	–0.02	0.03	–0.04	–0.02	0.03	–0.04	–0.02	0.03	–0.04
Gender	0.52	0.14	0.31***	0.10	0.12	0.06	0.02	0.11	0.01	–0.01	0.11	–0.00
Direct contact frequency	0.05	0.11	–0.04	–0.05	0.08	–0.05	–0.05	0.08	–0.05	–0.05	0.08	–0.05
Digital contact frequency	0.14	0.08	0.17	0.12	0.06	0.15	0.07	0.06	0.09	0.07	0.06	0.09
Need to belong				–0.13	0.06	–0.15*	–0.07	0.06	–0.13	–0.07	0.06	–0.08
ER—lack of clarity				–0.13	0.05	–0.21**	–0.08	0.05	–0.13	–0.08	0.05	–0.13
ER—low impulse control				0.02	0.05	0.02	–0.02	0.05	–0.02	–0.05	0.05	–0.07
ER—limited strategies				–0.33	0.07	–0.48***	–0.26	0.06	–0.38***	–0.25	0.06	–0.37***
Loneliness—isolation							–0.21	0.07	–0.28***	–0.20	0.07	–0.26**
Loneliness—proximity							–0.10	0.07	–10	–0.09	0.07	–0.10
Cyberbullying										0.49	0.25	0.12*
Δ*R*^2^				0.40***			0.07***			0.02*		
*R* ^2^	0.15***			0.54***			0.61***			0.63***		

ER, emotion regulation; coding: gender: 1 female, 2 male; contact frequency from 0 never to 6 almost daily/daily; need to belong from 1 does not apply at all to 6 applies completely, well-being and emotion regulation problems from 0 nearly never to 5 nearly always; loneliness from 1 does not apply at all to 5 applies completely; cyberbullying from 0 never to 5 nearly daily. *p < 0.05, **p < 0.01, ***p < 0.001; N = 129.

Of the control variables, only gender predicted well-being positively in the first step (male participants reported higher well-being). The need to belong, emotion regulation—lack of clarity and emotion regulation—limited strategies were negative predictors of well-being, but when loneliness was introduced in step 3, only emotion regulation—limited strategies remained significant. The loneliness subscale “isolation” was a negative predictor, but the proximity subscale was not. In the fourth and last step, and contrary to hypothesis 2 that expected a negative relation, cyberbullying predicted well-being positively and was a significant predictor over and above all other mentioned variables.

**FIGURE 1:**
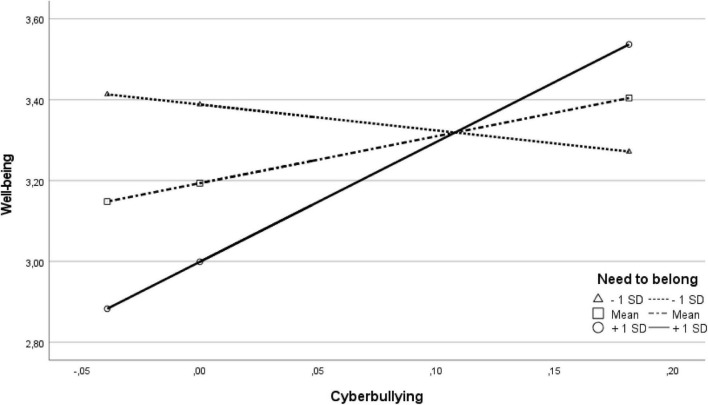
Visualization of the relation of cyberbullying and well-being for levels of need to belong.

Subsequently, we tested whether the need to belong or emotion regulation problems moderated the association between cyberbullying and well-being using the PROCESS macro in SPSS. The results for emotion regulation problems were not significant, thus contrary to hypothesis 3, emotion regulation problems did not moderate the relation between cyberbullying and well-being (interaction term for emotion regulation—lack of clarity: *B* = –0.45, *SE(B)* = 0.24, *t* = –1.84, *p* = 0.068, 95%*CI* [–0.92; 0.03]; emotion regulation—low impulse control: *B* = –0.53, *SE(B)* = 0.34, *t* = –1.57, *p* = 0.120, 95%*CI* [–1.19; 0.14]; emotion regulation—limited strategies: *B* = –0.64, *SE(B)* = 0.35, *t* = –1.85, *p* = 0.0678, 95%*CI* [–1.33; 0.05]). However, the need to belong was a significant moderator with a small effect size for this relation, thus confirming hypothesis 4. Results are displayed in Table 3 and Figure 1. Under conditions of a high need to belong (*M* + 1 *SD*), the higher the level of cyberbullying, the higher was the level of well-being (*B* = 2.96, *SE(B)* = 1.11, *t* = 2.66, *p* = 0.009, 95%*CI* [0.76; 5.16]). When the need to belong was on an average level, higher levels of cyberbullying still were associated with higher levels of well-being (*B* = 1.16, *SE(B)* = 0.40, *t* = 2.92, *p* = 0.004, 95%*CI* [0.38; 1.94]). But when the need to belong was low (*M*—1 *SD*), the relation was no longer significant (*B* = –0.64, *SE(B)* = 0.69, *t* = –0.92, *p* = 0.357, 95%*CI* [–2.01; 0.73]).

**TABLE 3:** Moderation of the relation of cyberbullying and well-being by need to belong.

					95% CI for *B*	
	*B*	*SE*(*B*)	*t*	*p*	*LL*	*UL*
Constant	3.19	0.06	53.68	< 0.001	3.08	3.31
Cyberbullying	1.15	0.40	2.93	0.004	0.38	1.94
Need to belong	–0.23	0.07	–3.14	0.002	–0.37	–0.08
Cyberbullying × Need to belong	2.09	0.97	2.15	0.033	0.17	4.02
Δ*R*^2^ for interaction	*R*^2^ = 0.02, *F*(1, 133) = 4.63, *p* = 0.033					
*R* ^2^	*R*^2^ = 0.16, *F*(3, 133) = 8.50, *p* < 0.001					

*Both variables were mean centered before calculation of the interaction; N = 137.*

**FIGURE 2:**
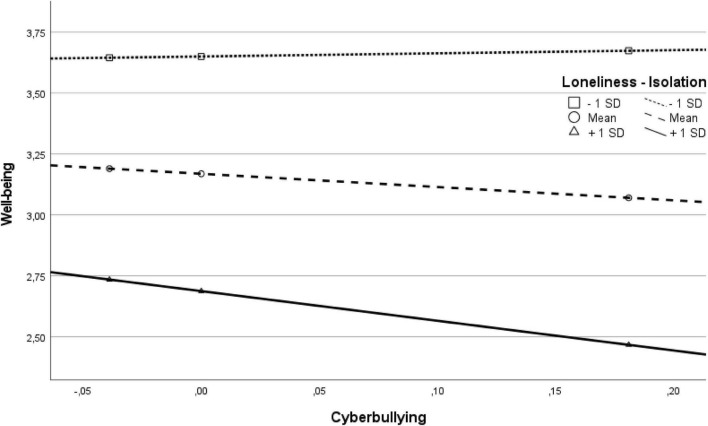
Visualization of the relation of cyberbullying and well-being for levels of loneliness—isolation.

In an exploratory analysis, we analyzed the moderating effect of both loneliness subscales lack of proximity and feelings of isolation on the association between cyberbullying and well-being using the PROCESS macro in SPSS. The result for loneliness—proximity was not significant; thus, proximity did not moderate the relation between cyberbullying and well-being (interaction term: *B* = 0.02, *SE(B)* = 0.56, *t* = 0.03, *p* = 0.977, 95%*CI* [–1.09; 1.13]). However, the loneliness—isolation was a significant moderator with a small effect size for this relation (see Table 4 and Figure 2). The levels of isolation (*M* + 1 *SD, M*, and *M*—1 *SD*, respectively) differed concerning the levels of well-being, however the slope of cyberbullying was not statistically different (all *p* > 0.05). Thus, the higher the level of isolation, the lower was the level of well-being (as indicated by the direct effect of isolation: *B* = –0.50, *SE(B)* = 0.05, *t* = –9.54, *p* < 0.001, 95%*CI* [–0.60; –0.40]).

**TABLE 4:** Moderation of the relation of cyberbullying and well-being by loneliness—isolation.

					95% CI for *B*	
	*B*	*SE*(*B*)	*t*	*p*	*LL*	*UL*
Constant	3.17	0.05	63.08	<0.001	3.07	3.27
Cyberbullying	–0.54	0.49	–1.12	0.266	–1.51	0.42
Loneliness—Isolation	–0.50	0.05	–9.54	<0.001	–0.60	–0.40
Cyberbullying × loneliness—isolation	–0.70	0.33	–2.13	0.035	–1.35	–0.05
Δ*R*^2^ for interaction	*R*^2^ = 0.02, *F*(1, 134) = 4.54, *p* = 0.034					
*R* ^2^	*R*^2^ = 0.65, *F*(3, 134) = 33.21, *p* < 0.001					

*Both variables were mean centered before calculation of the interaction; N = 138.*

## Discussion

The present study examined the role of cyberbullying, emotion regulation problems and need to belong in relation to well-being of adolescents in times of social contact restrictions. Generally, the regression model could significantly explain 63% of variance of well-being. Thus, considerable parts of differences between persons regarding their well-being were predicted by feelings of isolation, emotion regulation problems and cyberbullying. In the context of contact restrictions, the well-being of adolescents is influenced by predictors encompassing affective, emotional regulation and antisocial behavioral aspects of human life.

Surprisingly, cyberbullying and loneliness were negatively related. We expected a positive relation, with cyberbullying to function as a (maladaptive) way to overcome loneliness and elicit reactions from others. However, the more loneliness, the less cyberbullying adolescents reported. Loneliness is not only characterized by a negative valence; it also is an emotion with a rather low arousal (comparable to sad, depressed or bored; Russell, 1980). It is therefore plausible that loneliness does not activate aggressive behavior online. In research, loneliness has been found more often in cybervictims, not cyberbullies, and as a consequence but not as a precursor of cyberbullying involvement (e.g., Olenik-Shemesh et al., 2012; Han et al., 2021). In a short-term longitudinal study by Schultze-Krumbholz et al. (2012) the authors were able to show, though, that in boys cyberbullying reduced feelings of loneliness at the second measurement occasion. Since the present study is cross-sectional, the relationship could also be interpreted the other way around: the more cyberbullying is perpetrated, the less lonely do the perpetrators feel, which would be in line with the results from Schultze-Krumbholz et al. (2012).

Concerning the relation between cyberbullying and well-being, previous research has largely found negative associations (Kowalski et al., 2014; Guo, 2016). We therefore expected this negative relation but found the contrary: cyberbullying predicted well-being positively. Interestingly, the bivariate relation of cyberbullying and well-being was not significant, only in the context of other predictors like the feelings of loneliness, did cyberbullying positively predict well-being. Apparently, cyberbullying is only related to a more positive well-being in the context of contact restrictions and feelings of isolation. Experiencing relief from a forced state of passivity by attacking others aggressively seems to have a slight positive association with well-being. It has to be remembered that the effect size is only small and its standard error comparably high, so the association of cyberbullying and well-being is not a strong relation for all participants.

In hypothesis 3 we expected that emotion regulation problems would moderate the relation from cyberbullying on well-being. This was not substantiated; although the subscales “lack of clarity” and “limited strategies” nearly reached the level of significance (*p* = 0.068 and *p* = 0.067), no subscale of emotion regulation problems was a significant moderator. Having problems with emotion regulation was not influential concerning the relation of cyberbullying and well-being. This could be due to a close bivariate relation of emotion regulation problems with well-being, but not with cyberbullying. This later finding is surprising, given that several studies found a negative relation of emotion regulation problems with cyberbullying perpetration (Baroncelli and Ciucci, 2014; Gianesini and Brighi, 2015; Den Hamer and Konijn, 2016; Arató et al., 2020, 2021). Possibly, because of the strain due to contact restrictions and distance learning, emotion regulation problems were widely spread in our sample (as indicated by high standard deviations: lack of clarity *M* = 2.40, *SD* = 1.27, impulse control problems *M* = 2.07, *SD* = 1.12, limited strategies *M* = 2.55, *SD* = 1.10), so the values did not co-vary systematically with the values of cyberbullying. Further research is necessary to analyze these relations again. Possibly, latent class or latent profile analyses might be able to find subgroups with specific patterns.

Concerning the need to belong, hypothesis 4 expected a moderation of the relation between cyberbullying and well-being. Indeed, the higher the need to belong, the stronger was the relation between cyberbullying and well-being. Thus, especially for those adolescents with a high need to belong (that might not be fulfilled completely in the pandemic) the association of cyberbullying and well-being got closer. The result that cyberbullying perpetration predicted well-being positively is rather surprising in the light of previous cyberbullying research showing negative psycho-social outcomes also for perpetrators (Kowalski et al., 2014; Guo, 2016; Ansary, 2020). The moderation analysis provides a hint at underlying processes: In times of contact restrictions, cyberbullying may be a way of coming into contact with or eliciting reactions from others especially for those who had a high need to belong. Also, resorting to cyberbullying may be a way to solve or carry out conflicts that may otherwise have been carried out in school. Since we did not assess who the targets were of this aggression, this is currently still speculation. Affiliation as a motive has been associated with proactive aggression in the past. Proactive or instrumental aggression uses aggressive behavior as a means to achieve a specific goal (Little et al., 2003). It is positively linked to peer rejection (being disliked by peers) and negatively linked to social preference (being liked by more peers than disliked; Card and Little, 2006). While social support by peers has been identified as a protective factor against cyberbullying perpetration (e.g., Fanti et al., 2012; Arató et al., 2021), it is known from offline bullying research that the pure number of friends increases the risk of being a bully (e.g., Barboza et al., 2009; Wang et al., 2009; Meland et al., 2010). Contact restrictions might impact the quality of social relationships and reduce their positive and protective effects.

Concerning the experience of loneliness, the current study distinguished between two subscales of loneliness: feelings of isolation and lack of proximity. Although we did not expect these subscales to function differentially, it was to our surprise that only isolation, but not proximity was significantly positively related to the need to belong and negatively to cyberbullying in bivariate correlations. Additionally, only isolation, but not proximity was a significant negative predictor of well-being in the regression analysis. Thus, it seemed that feelings of isolation were more relevant in the context of contact restrictions, and the lack of satisfying social contacts seemed to be more important than the mere physical closeness to other persons. One might argue that being in physical distance can be tolerated to a certain extent, but feeling socially isolated becomes more important and is related to the non-fulfillment of the need to belong. Comparable to the situation of being physically close to others, but feeling lonely, human beings can feel connected to others although being physically distant.

The current study has limitations that should be kept in mind when interpreting the described results. The sample is rather small, and self-selection could have influenced its composition, which limits the generalizability of the results. The study is of cross-sectional nature, thus not allowing for the analysis of causal influences and changes over time. Participants reported their cyberbullying perpetration, emotion regulation problems and well-being in self-reports, which might be influenced by processes of social desirability and self-images of well-being in such special times of a pandemic. Also, we assessed the respective individual extent of adolescents’ need to belong as a trait, but not in how far this need was actually fulfilled. Our results therefore refer to more general personality patterns while the answers might be influenced by the present exceptional situation. The societal circumstances of the data collection were very specific; school closures and contact restrictions are an extraordinary situation for youth that is difficult to re-analyze or replicate in later studies. However, we see in this specific situation also a strength of the study which sheds light on the relation of cyberbullying and well-being under physical distancing.

Based on the results of the present study, theoretical and practical implications for intervention and prevention of cyberbullying emerge. The results have shown that in times of school closures and contact restrictions cyberbullying has a positive impact on students’ well-being. Since the study did not investigate who the victims of the perpetrated aggressions were, nor what social influence the perpetrators may have been under, follow-up studies should investigate what character traits of victims and perpetrators may be significant for the cyberbullying attacks and whether the aggression occurred due to the unusual circumstances during the pandemic and or due to increased media consumption and too little support from teachers, family and friends. For young people, contacts with peers are of central importance for building deeper relationships, gaining self-confidence in a social context and being able to develop their own identity (Walper et al., 2021). If the lack of such relationships has led to cyberbullying being used as a maladaptive strategy to increase well-being, child and youth services as well as prevention and intervention programs for cyberbullying could help to create an important compensatory function to reduce stress and enable motivating activities and social contacts in a protected space (Hellfeldt et al., 2020; Walper et al., 2021). Flexible, empathetic approaches, such as group discussions in class or individual conversations with the teacher, which should also be adapted to teaching in the virtual space, and thus the opportunity to talk openly about the personal impact of the pandemic, may prove to be helpful in normalizing the experience and understanding and alleviating the concerns of many young adults at this time so that maladaptive strategies such as cyberbullying are no longer used to improve one’s well-being (Demkowicz et al., 2021).

## Data Availability Statement

The raw data supporting the conclusions of this article will be made available by the authors, without undue reservation.

## Ethics Statement

Ethical review and approval was not required for the study on human participants in accordance with the local legislation and institutional requirements. Written informed consent from the participants’ legal guardian/next of kin was not required to participate in this study in accordance with the national legislation and the institutional requirements.

## Author Contributions

JP, AS-K, and KL conceived of the study and participated in its design and coordination jointly. JP conducted the statistical analysis and drafted the manuscript. AS-K and KL helped to draft the manuscript. All authors read and approved the final manuscript.

## Conflict of Interest

The authors declare that the research was conducted in the absence of any commercial or financial relationships that could be construed as a potential conflict of interest.

## Publisher’s Note

All claims expressed in this article are solely those of the authors and do not necessarily represent those of their affiliated organizations, or those of the publisher, the editors and the reviewers. Any product that may be evaluated in this article, or claim that may be made by its manufacturer, is not guaranteed or endorsed by the publisher.
